# The PP1 regulator PPP1R2 coordinately regulates AURKA and PP1 to control centrosome phosphorylation and maintain central spindle architecture

**DOI:** 10.1186/s12860-020-00327-5

**Published:** 2020-11-25

**Authors:** Alan-Michael Bresch, Nadiya Yerich, Rong Wang, Ann O. Sperry

**Affiliations:** 1 Department of Anatomy and Cell Biology, Brody School of Medicine, East Carolina University, Greenville, NC 27834 USA; 2 University of North Carolina School of Medicine, Chapel Hill, NC 27599 USA

**Keywords:** PPP1R2, Aurora a (AURKA), Protein phosphatase 1 (PP1), Centrosome, Cytokinesis

## Abstract

**Background:**

Maintenance of centrosome number in cells is essential for accurate distribution of chromosomes at mitosis and is dependent on both proper centrosome duplication during interphase and their accurate distribution to daughter cells at cytokinesis. Two essential regulators of cell cycle progression are protein phosphatase 1 (PP1) and Aurora A kinase (AURKA), and their activities are each regulated by the PP1 regulatory subunit, protein phosphatase 1 regulatory subunit 2 (PPP1R2). We observed an increase in centrosome number after overexpression of these proteins in cells. Each of these proteins is found on the midbody in telophase and overexpression of PPP1R2 and its mutants increased cell ploidy and disrupted cytokinesis. This suggests that the increase in centrosome number we observed in PPP1R2 overexpressing cells was a consequence of errors in cell division. Furthermore, overexpression of PPP1R2 and its mutants increased midbody length and disrupted midbody architecture. Additionally, we show that overexpression of PPP1R2 alters activity of AURKA and PP1 and their phosphorylation state at the centrosome.

**Results:**

Overexpression of PPP1R2 caused an increase in the frequency of supernumerary centrosomes in cells corresponding to aberrant cytokinesis reflected by increased nuclear content and cellular ploidy. Furthermore, AURKA, PP1, phospho PPP1R2, and PPP1R2 were all localized to the midbody at telophase, and PP1 localization there was dependent on binding of PPP1R2 with PP1 and AURKA as well as its phosphorylation state. Additionally, overexpression of both PPP1R2 and its C-terminal AURKA binding site altered enzymatic activity of AURKA and PP1 at the centrosome and disrupted central spindle structure.

**Conclusions:**

Results from our study reveal the involvement of PPP1R2 in coordinating PP1 and AURKA activity during cytokinesis. Overexpression of PPP1R2 or its mutants disrupted the midbody at cytokinesis causing accumulation of centrosomes in cells. PPP1R2 recruited PP1 to the midbody and interference with its targeting resulted in elongated and severely disrupted central spindles supporting an important role for PPP1R2 in cytokinesis.

**Supplementary Information:**

The online version contains supplementary material available at 10.1186/s12860-020-00327-5.

## Background

The centrosome is a nonmembrane-bounded cytoplasmic organelle that nucleates radial microtubule arrays in both interphase and mitosis. Centrosome function establishes cell polarity, assembles the mitotic spindle to faithfully segregate chromosomes at mitosis, and aligns the midbody during cell division [1, 2]. Centrioles duplicate once per cell cycle at S-phase, separate, undergo maturation during G2-phase characterized by the recruitment of proteins to the pericentriolar matrix (PCM), and then nucleate microtubules to form the mitotic spindle. It is essential that the cell maintain a normal number of centrosomes, a process dependent on proper duplication of centrosomes along with their accurate distribution at cell division. Failure of cytokinesis, due to chromosomal abnormalities, mitotic checkpoint dysregulation, or malfunction of the numerous proteins involved in cytokinesis [3], among others, leads to supernumerary centrosomes [4]. Indeed, overexpression of AURKA kinase (AURKA) overrides the mitotic checkpoint to cause cytokinesis failure and tetraploidization [5, 6]. Aberrant centrosome number leads to multipolar spindles, improper cell division, aneuploidy, and is strongly correlated with cancer [7, 8].

Cell cycle progression is dependent, in part, on the activity of Aurora A kinase (AURKA) and protein phosphatase 1 (PP1) [9]. PP1 regulates a myriad of cellular processes some tied to centrosome biology including mitosis, cytokinesis, and the cell cycle [10–12]. PP1’s roles are specified by binding of its catalytic subunit to as many as 200 different regulatory subunits [11, 13]. Interactions with specific regulatory subunits and centrosome scaffolding proteins recruit PP1 to the centrosome and midbody [10, 12, 14].

AURKA is a highly conserved serine-threonine kinase that regulates centrosome maturation, spindle formation, and cytokinesis [15]. Loss of AURKA function results in monopolar spindles and bipolar spindles with two centrosomes at one pole, indicating that centrosomes fail to segregate in the absence of AURKA [16, 17]. AURKA overexpression causes centrosome multiplication through failure of cytokinesis in cultured mammalian cells and occurs along with centrosome amplification in cancer [6, 18–20]. PP1 and its subunits counteract activities of the mitotic kinases AURKA and AURKB to maintain proper spindle formation and chromosome segregation during mitosis [21–25]. Centrosome number depends on regulation of both centrosome duplication and cytokinesis; both events rely on the balanced activities of protein phosphatases and kinases [1, 26, 27].

One PP1 regulatory subunit, PPP1R2, was identified as a heat-stable inhibitor of PP1 [28, 29]. PPP1R2 activity peaks during mitosis, where it regulates centrosome separation, chromosome segregation, and cytokinesis [15, 22, 30, 31]. The PP1-PPP1R2 complex induces centrosome separation prior to mitosis through association with ‘Never in Mitosis Kinase 2’ (NEK2) [27, 32]. PPP1R2 is a bifunctional protein – in addition to inhibiting PP1, PPP1R2 also regulates the G2/M transition by binding and activating AURKA [23, 30].

PPP1R2 interacts with both AURKA and PP1, enzymes that regulate cytokinesis [33, 34]; therefore, we hypothesized that overexpression of PPP1R2 would interact with AURKA and PPI to interfere with cytokinesis and cause supernumerary centrosomes. The aims of this study were: 1) to determine whether PPP1R2 functionally interacts with PP1 and AURKA and 2) to investigate a role for PPP1R2 in cytokinesis. Our results indicate PP1 and PPP1R2 oppose AURKA activity to prevent the accumulation of centrosomes in AURKA overexpressing cells. We show that the C-terminus of PPP1R2 regulates the activity of AURKA and PP1. The supernumerary centrosomes seen in PPP1R2 overexpressing cells suggested a failure of cytokinesis similar to that seen in cells overexpressing AURKA [5, 6]. As expected for a protein important for cytokinesis, overexpression of PPP1R2 increased nuclear content and cell ploidy and was localized, along with AURKA and PP1, to the midbody at telophase. Overexpression of PPP1R2 or its phosphomimetic enhanced targeting of PP1 to the midbody while overexpression of PPP1R2 truncation mutants caused elongated and distorted central spindles. Together, our findings demonstrate that both PPP1R2 phosphorylation and its interaction with AURKA and PP1 are necessary to direct PP1 to the midbody in an ideal location to regulate events prior to abscission.

## Results

### PPP1R2, AURKA, and PP1 interaction affects supernumerary centrosomes

**Fig. 1:**
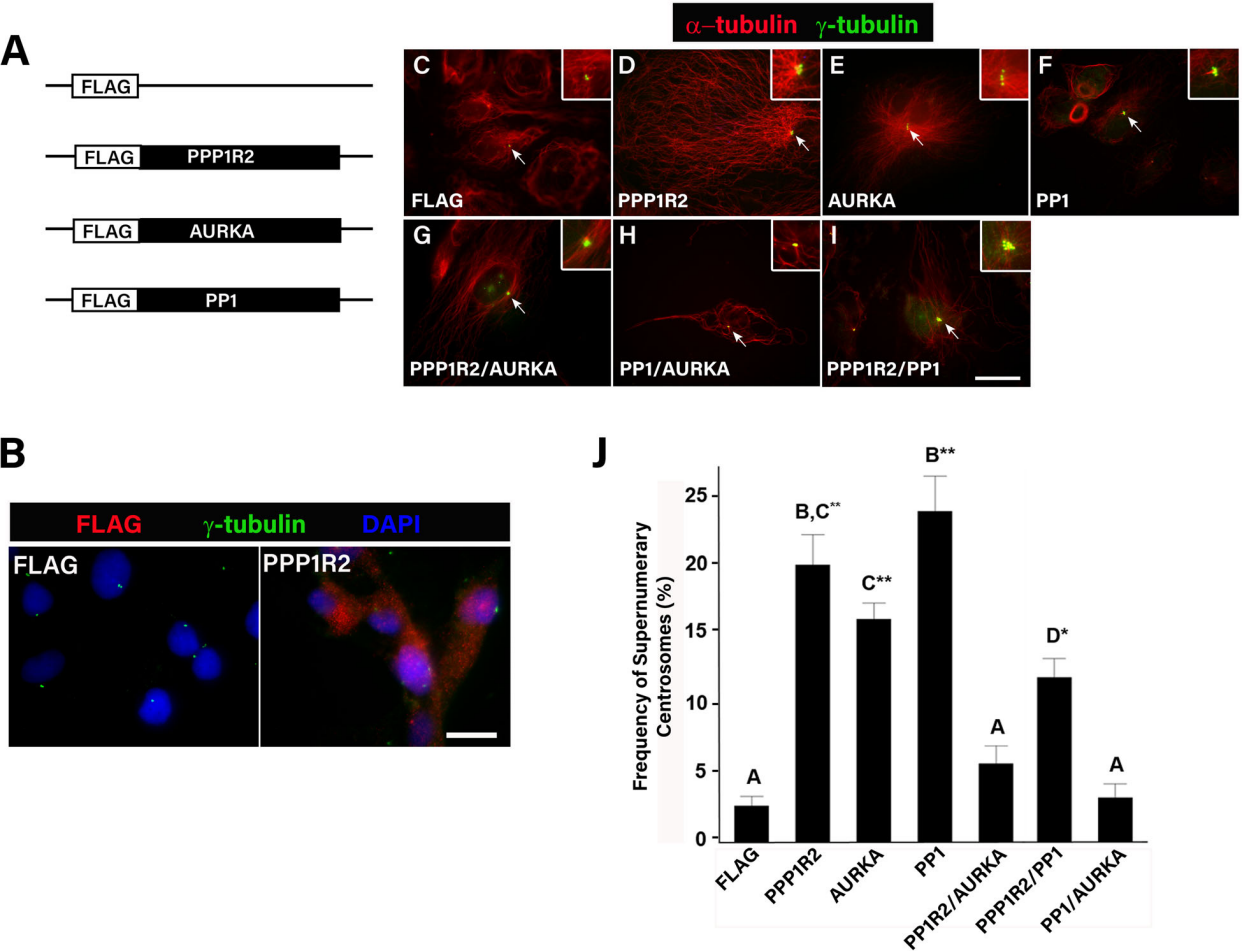
PPP1R2 affects centrosome number through interaction with AURKA and PP1. **a-b** Schematic of constructs used for transfection and representative transfection. **c-i** ARPE-19 cells were transfected either singly or in combination with plasmids expressing PPP1R2, AURKA, PP1 and empty vector as control (FLAG). Transfected cells were stained for γ-tubulin (green) and α-tubulin (red) and centrosomes were counted in a minimum of 100 cells for each treatment group in three replicates. Insets magnify cellular regions containing centrosomes. Size bar equals 10 μm. **j** Graphical representation of the frequency of supernumerary centrosomes in cells transfected with each of the indicated plasmids individually or in combination. Statistically significant differences (*p* < 0.05) between groups are indicated by differing letter notations above the bars and error bars represent standard error of the mean. Statistically significant differences are indicated with asterisks (* = *p* ≤ 0.01, ** = *p* ≤ 0.001) compared to control

PPP1R2 has been shown to regulate cytokinesis through interaction with Aurora B (AURKB) [24] and PPP1R2 regulates both PP1 [28, 29] and AURKA [23] that have been previously shown to regulate cytokinesis [6, 10, 12, 35]. To better define if PPP1R2 interacts with AURKA and PP1 to cause supernumerary centrosomes, we overexpressed epitope-tagged proteins in ~ 85% of ARPE-19 cells (Fig. 1a-b and Additional file 1). Overexpression of PPP1R2 as well as AURKA and PP1 caused supernumerary centrosomes, with some cells having as many as 6 centrosomes, visualized as γ-tubulin puncta (Fig. 1d-f, inset) compared to 1–2 found in empty vector controls (Fig. 1c, inset). The frequency of supernumerary centrosomes in cells overexpressing PPP1R2 increased 8-fold compared to cells transfected with empty vector (*p* ≤ 0.0001) (Fig. 1j). Co-overexpression of AURKA and PPP1R2 restored supernumerary centrosome frequencies to control levels (Fig. 1g inset, j). In contrast, co-overexpression of PP1 and PPP1R2 only partially restored supernumerary centrosome frequency (Fig. 1i inset, j). Overexpression of PP1 and AURKA together in cells did not increase centrosome number compared to controls, in contrast to the effect of AURKA and PP1 alone (Fig. 1e-f inset, j). These results reveal the importance of interaction between PP1 and AURKA as well as between PPP1R2 and both AURKA and PP1 to prevent the accumulation of centrosomes in cells.

### PPP1R2 interaction with both PP1 and AURKA increases centrosome number and is phosphorylation dependent

**Fig. 2:**
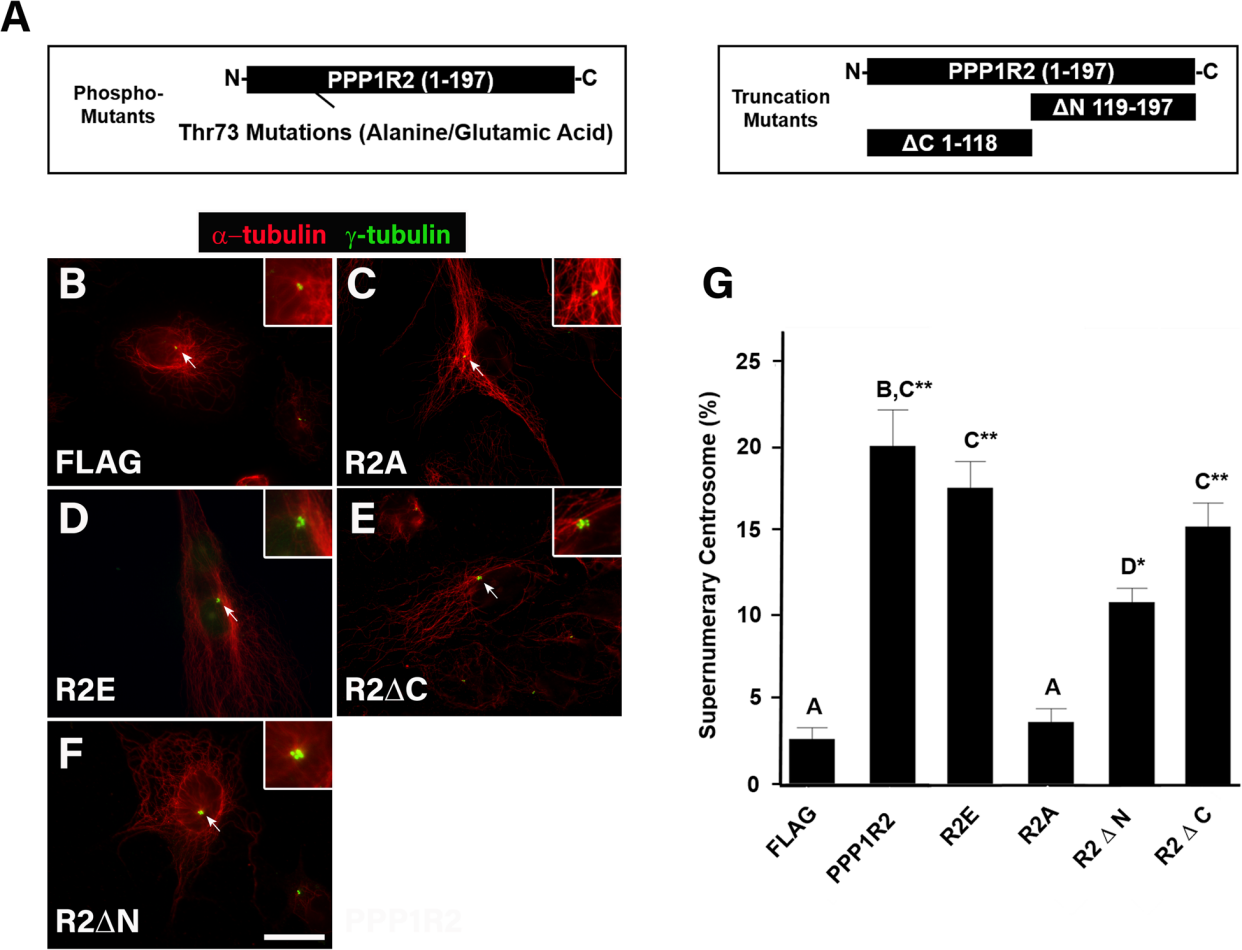
PPP1R2 interaction with both PP1 and AURKA affects centrosome number and is phosphorylation dependent. **a** Schematics of the PPP1R2 mutants used for transfection. The left schematic shows the position of phosphorylation site mutants involving the Thr73 residue including both the threonine to alanine phosphonull mutation PPP1R2A (R2A) and threonine to glutamic acid phosphomimetic mutation PPP1R2E (R2E). The right schematic indicates position of PPP1R2 truncations which included PPP1R2 plasmids truncated at either the N-terminus PPP1R2ΔN (R2ΔN), deleting the PP1 binding site, or the C-terminus PPP1R2ΔC (R2ΔC), removing the AURKA binding site. These mutants were tested for their affect on centrosome number (**c-f**). Cells were transfected with the indicated proteins, fixed, and stained with anti-γ-tubulin (green) and anti-α-tubulin (red). Size bar equals 10 μm. Insets show the region containing centrosomes. **g** Quantitation of the percentage of cells with supernumerary centrosomes after transfection with the indicated constructs. Statistically significant differences among groups are indicated by differing letter notations above the bars at *p* ≤ 0.05 (*p ≤ 0.01, **p ≤ 0.001)

Glycogen synthase kinase-3β is known to inactivate PPP1R2 by phosphorylating threonine 72, thereby activating PP1 [29, 36, 37]. In order to test the relevance of this modification to PPP1R2 function, we created a homologous mutation in human PPP1R2, at Thr73 from threonine to either alanine (R2A, which cannot be phosphorylated) or glutamic acid (R2E, a phosphomimetic) as shown in Fig. 2a (left). These plasmids were transfected into ARPE-19 cells (Fig. 2c-d) to test whether induction of supernumerary centrosomes by PPP1R2 was dependent upon its phosphorylation state. Compared to control cells (Fig. 2b, inset), overexpression of the PPP1R2 R2A (R2A) mutant did not increase centrosome numbers (Fig. 2c inset, g). In contrast, overexpression of the PPP1 R2E (R2E) mutant increased the frequency of supernumerary centrosomes ~ 7-fold (*p* ≤ 0.0001; Fig. 2d inset, g).

PP1 and AURKA bind to separate domains on the PPP1R2 protein; PP1 binds to the N-terminal region (aa 1–118), while AURKA binds to the C-terminal region (aa 119–197) (Fig. 2a, right) [23, 28]. Therefore, we tested the effect of deletion of these binding domains on induction of supernumerary centrosomes. This was accomplished by transfecting PPP1R2 truncation mutants lacking either the PP1 (R2ΔN; Fig. 2a, right) or the AURKA binding site (R2ΔC; Fig. 2a, right), respectively. All proteins were detectable by ELISA after transfection, except for R2ΔC that lacked an epitope tag (Additional file 1). Deletion of either the PP1 or the AURKA binding site on PPP1R2 reduced the frequency of supernumerary centrosomes compared to full length PPP1R2 (Fig. 2e-g), revealing that interaction with both PP1 and AURKA is necessary to cause supernumerary centrosomes.

### PPP1R2 overexpression increased cell ploidy and accumulation of cells with enlarged nuclei

**Fig. 3:**
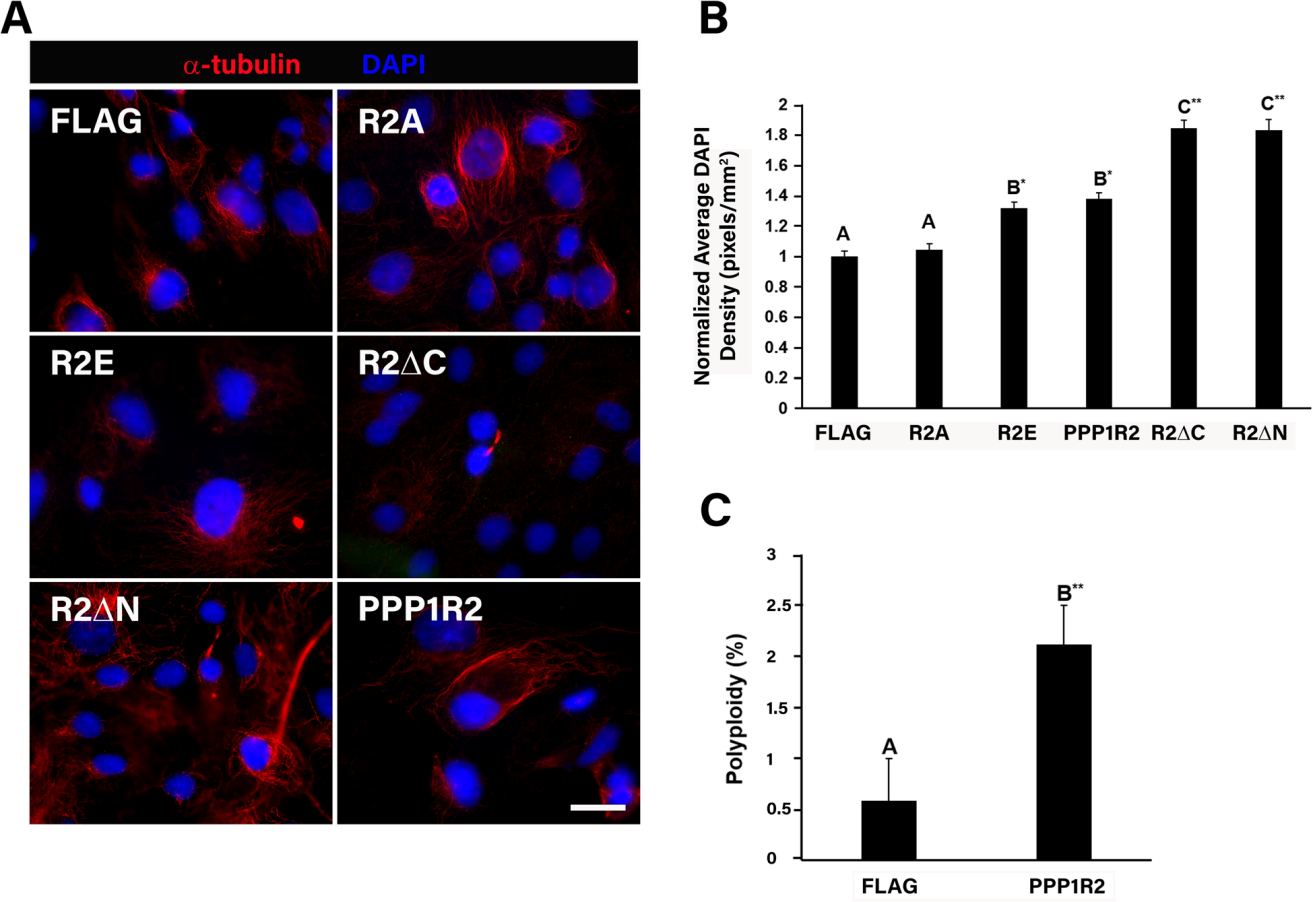
PPP1R2 overexpression increased cell ploidy and accumulation of cells with enlarged nuclei. **a** Cells were transfected with the indicated FLAG-tagged fusion proteins, fixed and stained for α-tubulin to detect microtubules (red), and DAPI to stain the nucleus (blue). Size bars equal 10 μm. **b** Quantitation of DAPI intensity after transfection with the indicated constructs and normalized to the control. **c** Quantitation of cells with increased ploidy after transfection with PPP1R2. Statistically significant differences (*p* ≤ 0.05) between experimental groups and empty vector control are indicated by differing letters above the bars and asterisks indicate *p ≤ 0.01 and **p ≤ 0.001

AURKA overexpression was previously shown to induce tetraploidization through cytokinesis failure [25–27]. Overexpression of PPP1R2 increased the number of cells with increased DNA content, as compared to empty vector controls (*p* ≤ 0.001, Fig. 3a-b). Overexpression of the PPP1R2 R2E phosphomimetic mutant also significantly increased cellular DNA content (*p* ≤ 0.01) as compared to cells overexpressing PPP1R2 R2A (R2A) or the empty vector control (Fig. 3a-b). The number of cells with increased ploidy quadrupled following PPP1R2 overexpression consistent with the observed increase in DNA content (Fig. 3c). Both R2 C- and N- terminal truncation mutant overexpression resulted in the most significant increase in nuclear content (Fig. 3b). Our result is similar to that seen when AURKA is overexpressed [6]; therefore, we investigated whether PPP1R2 causes an increase in chromosome number through an effect on cytokinesis.

### PPP1R2, pPP1R2, PP1, and AURKA are localized to the midbody during cytokinesis

**Fig. 4:**
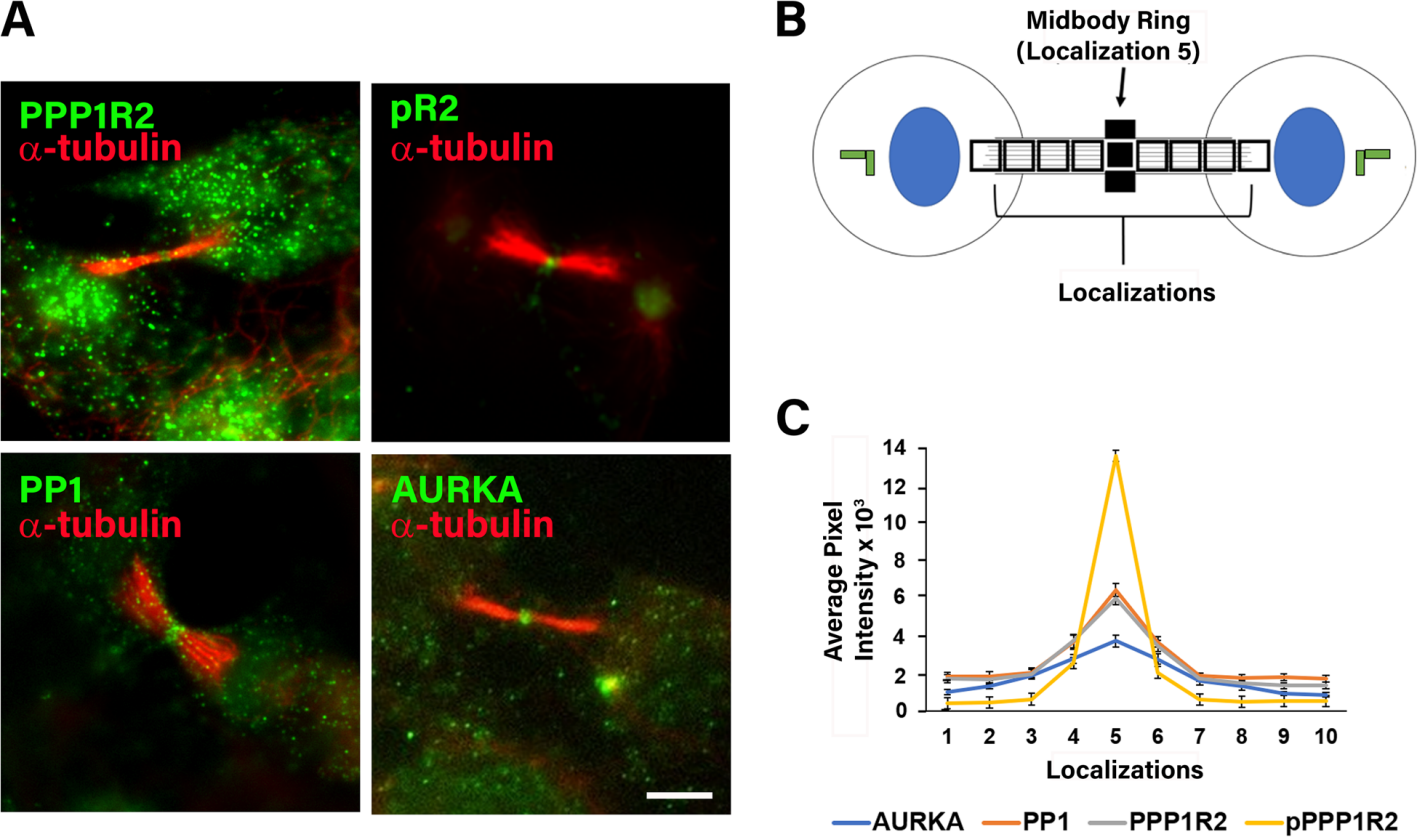
PP1, PPP1R2, pR2, and AURKA are localized at the midbody. **a** ARPE-19 cells were fixed and stained for the indicated endogenous proteins (green) localized relative to α-tubulin of the central spindle (red). **b** Average intensity from the green channel along the central spindle was measured in 10–0.87 μm^2^ square regions spanning the length of the central spindle in 30 dividing cells and is plotted in (**c**). Size bar equals 5 μm. Asterisks indicate statistical significance compared to control (*p ≤ 0.001, **p ≤ 0.001) and error bars represent standard error of the mean

Given our data and previously established roles of AURKA and PP1 in cytokinesis, we next investigated localization of AURKA, PP1, PPP1R2, and phospho PPP1R2 in dividing ARPE-19 cells. The midbody and central spindle are distinct structures formed during cytokinesis, and we established a scoring system to quantify fluorescent levels of proteins across each structure. AURKA, PP1, pR2 (phospho-PPP1R2), and PPP1R2, were each detected at telophase on the midbody (Fig. 4a). Fluorescent localization of AURKA, PP1, PPP1R2 and pR2 was quantified along the midbody in equal-sized boxes (Fig. 4b). PPP1R2, pR2, PP1, and AURKA were all positioned at the midbody (Fig. 4c), consistent with a shared role in cytokinesis. Interestingly, pR2 localization was more enriched at the midbody than the entire pool of PPP1R2 (Fig. 4a).

### PPP1R2 targets PP1 to the midbody

**Fig. 5:**
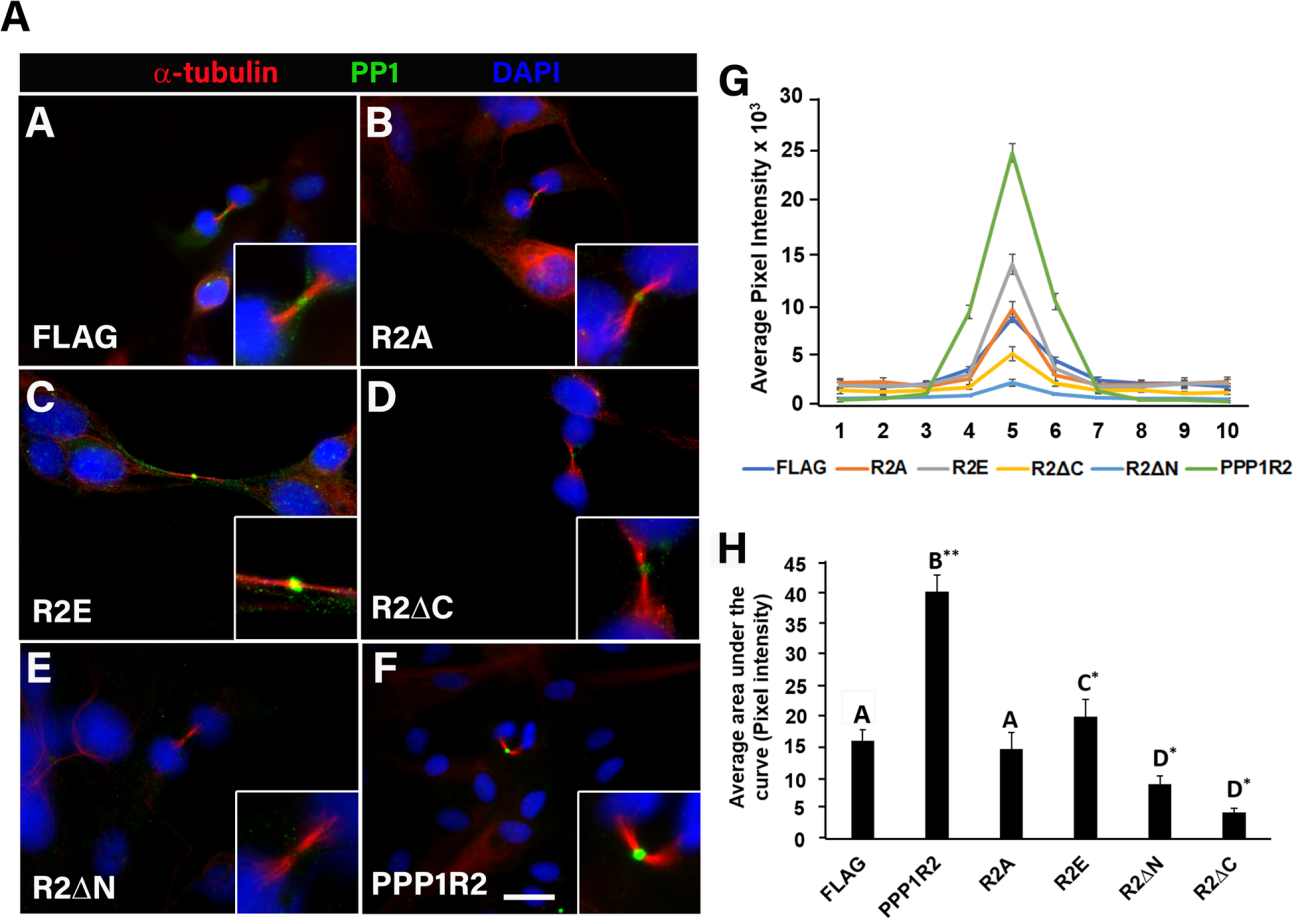
PPP1R2 targets PP1 to the midbody. **a-f** ARPE-19 cells were transfected, fixed, and labeled for PP1 (green) to determine the localization of endogenous PP1 along the central spindle (red) following overexpression of PPP1R2 mutants: a phosphonull Thr73 mutant PPP1R2A (R2A), a phosphomimetic Thr73 mutant PPP1R2E (R2E), a C-terminal truncation PPP1R2ΔC (R2ΔC), and an N-terminal truncation PPP1R2ΔN (R2ΔN). Average intensity from the green channel was measured along the central spindle in equal sized squares (*n* = 30 dividing cells) and is plotted in (**g**). The area under the curve was calculated for total PP1 localization intensity throughout the midbody’s structure (**h**). Error bars represent standard error of the mean. Statistically significant differences (*p* ≤ 0.05) between experimental groups and empty vector control are indicated by differing letters above the bars and asterisks indicate *p ≤ 0.01 and **p ≤ 0.001

We next investigated if full length PPP1R2 could increase PP1 location at the midbody and if PPP1R2 mutants could interfere with this targeting. We transfected ARPE-19 cells with full length PPP1R2 or the mutants and measured endogenous PP1 localization at the midbody. PPP1R2 overexpression resulted in a significant (*p* < .001) increase in localization of PP1 at the midbody (Fig. 5f inset, G-H). Interestingly, PPP1R2 R2A (R2A) (phosphonull) overexpression had no significant effect on PP1 localization when compared to empty vector control (Fig. 5a-b inset, g-h). In contrast, PPP1R2 R2E (R2E) (phosphomimetic) overexpression significantly (*p* < .01) enhanced PP1 localization at the midbody (Fig. 5c inset, g-h) compared to both empty vector control and R2A mutant overexpression. This difference suggests PPP1R2 phosphorylation directs PP1 recruitment to the midbody. R2ΔN and R2ΔC overexpression significantly reduced PP1 midbody localization, as compared to empty vector control (*p* < 0.001) (Fig. 5d-e, g-h) Overall, both N-terminal and C-terminal domains were necessary for PP1 recruitment to the midbody at telophase and phosphorylated PPP1R2 is critical to this process.

### PPP1R2 mutant overexpression altered both central spindle length and structure

**Fig. 6:**
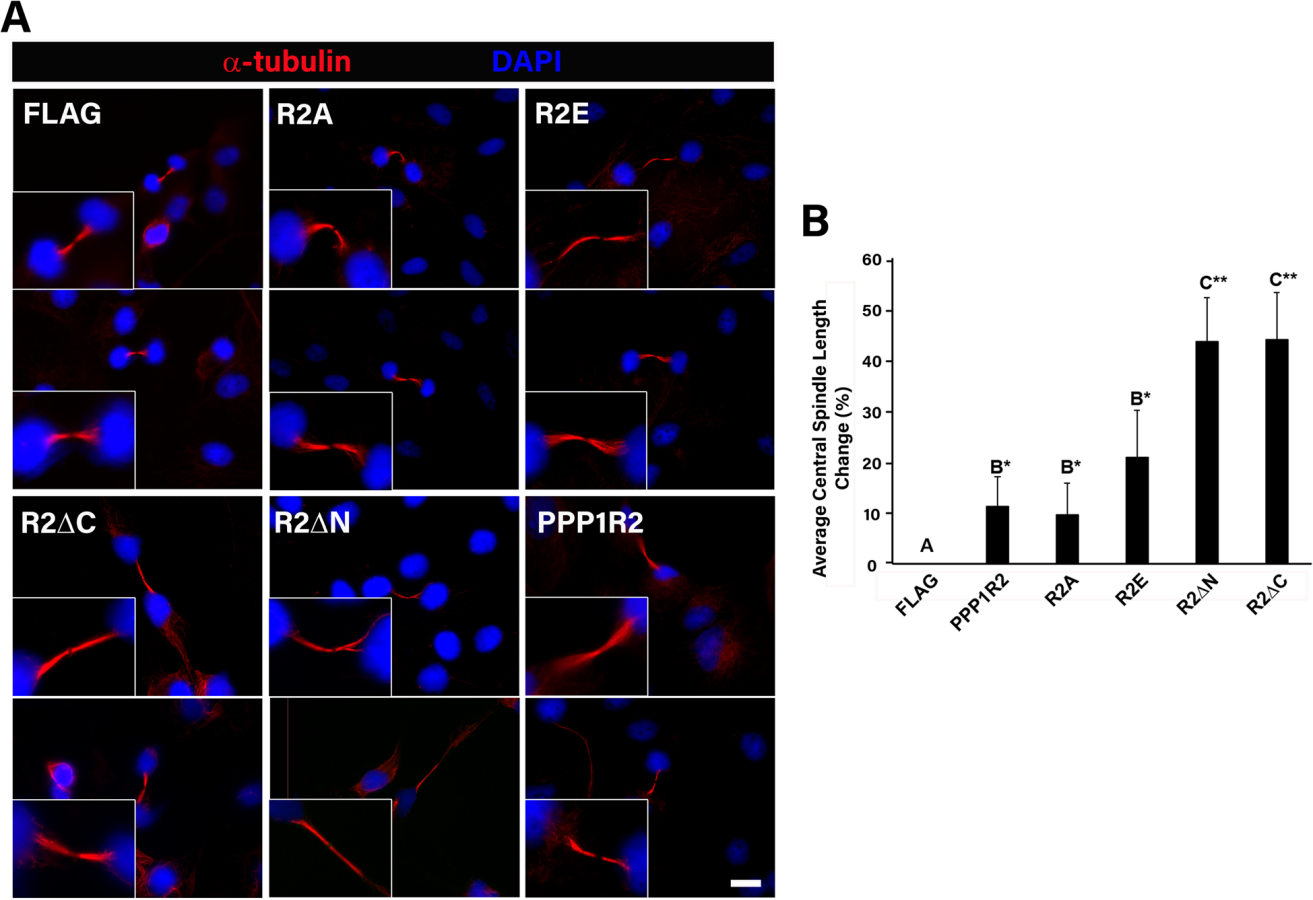
PPP1R2 mutant overexpression increased midbody length. **a** ARPE-19 cells were transfected with FLAG, PPP1R2, and PPP1R2 mutants (R2A, R2E, R2ΔC, R2ΔN), fixed, and labeled with DAPI (blue) as well as α-tubulin (red) to stain the central spindle. Size bar equals 5 μm. **b** Midbody length was quantified using Metamorph software and averages were calculated within three biological replicates (*n* ≥ 30 dividing cells). Statistically significant differences (*p* ≤ 0.05) between experimental groups and empty vector control are indicated by differing letters above the bars and asterisks indicate *p ≤ 0.01 and **p ≤ 0.001

**Fig. 7:**
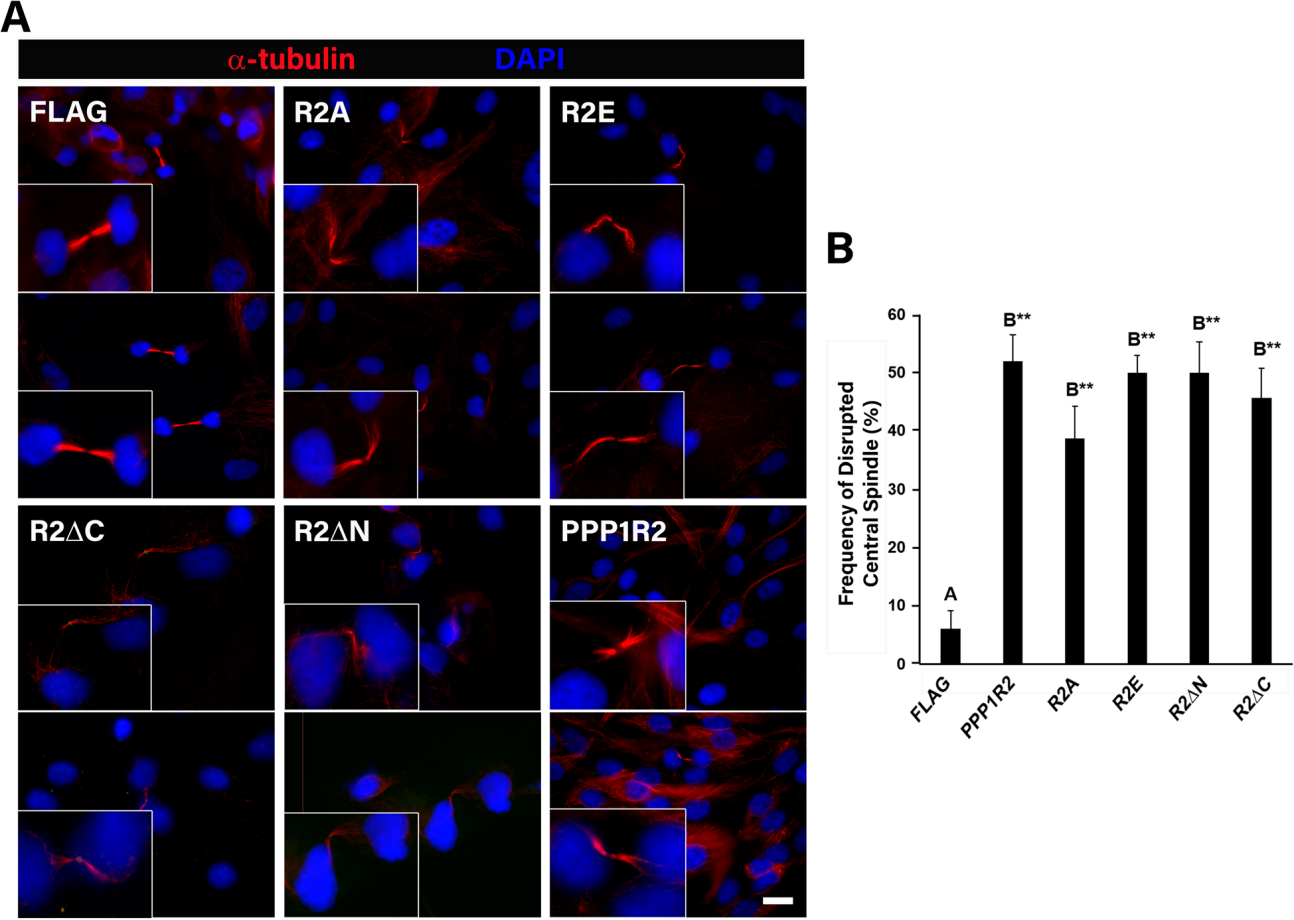
PPP1R2 mutant overexpression increased frequency of disrupted central spindle structure. **a** ARPE-19 cells were transfected with FLAG, PPP1R2, and PPP1R2 mutants (R2A, R2E, R2ΔC, R2ΔN), fixed, and labeled with DAPI (blue) as well as α-tubulin (red) to stain the central spindle. Size bar equals 5 μm. **b** Disrupted midbody frequency was quantified using Metamorph software and averages were calculated for three biological replicates (n ≥ 30 dividing cells). Statistically significant differences (*p* ≤ 0.05) between experimental groups and empty vector control are indicated by differing letters above the bars and asterisks indicate *p ≤ 0.01 and **p ≤ 0.001

We investigated if overexpression of PPP1R2 and PPP1R2 mutants affected central spindle length and observed elongated central spindles in cells overexpressing these constructs (Fig. 6). Cells overexpressing PPP1R2 or its mutants displayed central spindles that were on average > 20% longer than controls. This was observed in both phosphomimetic as well as truncated mutant overexpression (Fig. 6a-b). Truncation mutants resulted in significantly longer central spindles compared to all other treatment groups (Fig. 6b, *p* < 0.001). Additionally, overexpression of PPP1R2 mutants significantly increased the frequency of abnormal central spindle morphology in transfected cells (Fig. 7a-b, *p* < 0.01). Abnormal central spindle morphology was defined as those with either an irregular tortuous structure or unraveled microtubules; these phenotypes were rarely seen in control treated cells. Although the frequency of disrupted central spindles increased to the same level as full length PPP1R2 for each of the mutants compared to control (Fig. 7b), the severity of misshapen central spindles was most pronounced in cells overexpressing the truncation mutants (Fig. 7a, R2ΔC and R2ΔN). The high degree of disruption seen in the truncation mutants correlated with an increase in central spindle length compared to other constructs (Fig. 6b). We propose that the increase in central spindle length contributes to distortion of this structure in the truncation mutants. Microtubule bundling was profoundly disrupted in the central spindle in the region closest to DNA in cells overexpressing each PPP1R2 mutant. Altogether this data suggests that PPP1R2 has a role in maintaining central spindle architecture.

### The effect of PPP1R2 on PP1 and AURKA activity at the centrosome was dependent on its C-terminus

**Fig. 8:**
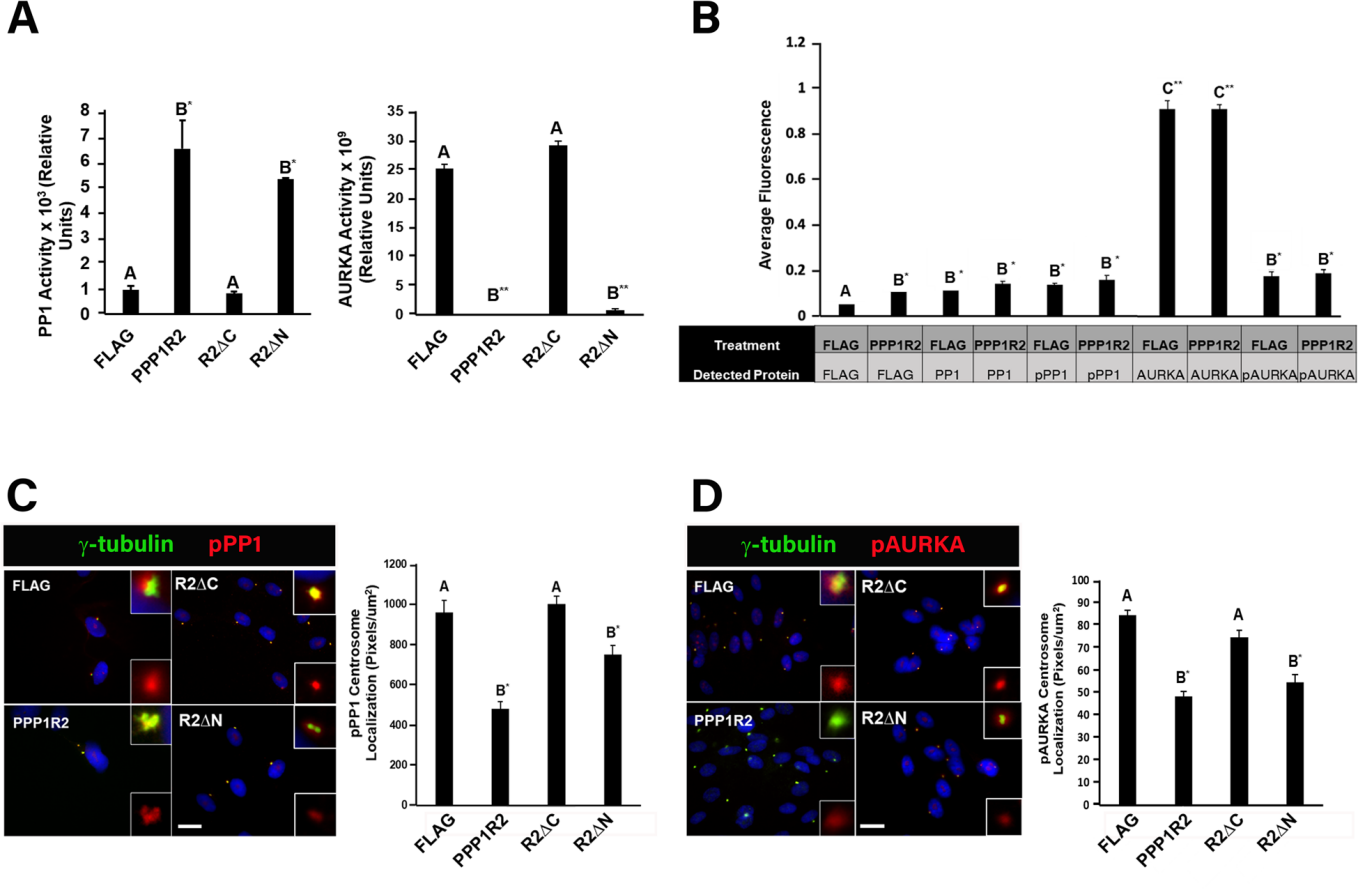
The effect of PPP1R2 on PP1 and AURKA activity at the centrosome was dependent on its C-terminus. **a** A graphical representation of the PP1 and AURKA activity of cells expressing each construct is shown. **b** Protein expression and phosphorylation levels of proteins indicated by the table below the graph were measured using ELISA. Cells were transfected with the indicated constructs, fixed, and labeled for γ-tubulin (red) and either (**c**) phosphorylated PP1 (pPP1, green) or (**d**) phosphorylated AURKA (pAURKA, green) antibodies. pPP1 and pAURKA levels at the centrosome were quantified using Metamorph software and are displayed as graphs in C and D. Statistically significant differences (*p* ≤ 0.05) between experimental groups and empty vector control are indicated by differing letters above the bars and asterisks indicate *p ≤ 0.01 and **p ≤ 0.001

PPP1R2 overexpression stimulated PP1 activity, while at the same time reducing AURKA activity to undetectable levels (Fig. 8a). These effects were independent of protein amount or phosphorylation state (Fig. 8b). Overexpression of PPP1R2 truncated at its C-terminal domain (R2ΔC) did not change the activity of either PP1 or AURKA relative to control. In contrast, deletion of its N-terminus had the opposite effect by increasing PP1 activity to a level statistically similar to full length PPPR2 while at the same time abolishing AUKA, is necessary and sufficient to stimulate PP1 activity and inhibit AURKA activity.

Consistent with the observed changes in global enzyme activity after PPP1R2 overexpression, both AURKA and PP1 were less phosphorylated at the centrosome (Figure C-D) when PPP1R2 was overexpressed, corresponding to increased PP1 activity and reduced AURKA activity at the centrosome. This supports our proposal that PPP1R2 overexpression increased PP1 activity through its repression of AURKA activity resulting in a loss of phosphorylation at the centrosome. In addition, only PPP1R2 containing the C-terminus caused a decrease in pPP1 and pAURKA levels at the centrosome confirming that the C-terminus of PPP1R2 is required to inhibit AURKA and stimulate PP1 activity (Fig. 8c-d).

## Discussion

AURKA regulates cell division, in part, through establishment of the bipolar spindle, and cytokinesis [16, 17, 35]. AURKA overexpression causes centrosomes to accumulate in cells through failure of cytokinesis [6, 18–20]. AURKA activity is opposed by PP1 through a negative feedback loop and this interaction is required for proper cell division. Disruption of binding of PP1 to AURKA leads to misalignment of chromosomes at metaphase [21]. The PP1 regulator PPP1R2 binds both AURKA and PP1 to positively and negatively regulate these enzymes [23]. These interactions are consistent with our results that truncation of either the PP1 or the AURK binding sites partially reduced the level of supernumerary centrosomes compared to the full-length PPP1R2 (Fig. 2g).

Here we demonstrate that, like the negative feedback relationship between AURKA and PP1, PPP1R2 opposes the ability of AURKA to cause multiple centrosomes in cells. Our finding that PPP1R2 overexpression countered the effect of AURKA to increase centrosome number suggests that PPP1R2 inhibits AURKA; a finding that contradicts a previous study [23]. This difference may be a consequence of environment; our studies were conducted in live cells, while that study used recombinant proteins in vitro [23] to show stimulation of AURKA activity by PPP1R2.

Although PPP1R2 overexpression with AURKA reduced centrosome number to control levels, it did not have the same effect when overexpressed with PP1. Instead, supernumerary centrosome frequency was reduced by about half in double transfectants compared to PP1 alone (Fig. 1j). We suspect this intermediate effect on centrosome number is due to an inhibition of PP1 by PPP1R2 to lessen phosphatase activity available to counteract AURKA. PPP1R2 is a substrate of PP1 [13]; therefore, these two proteins could form a negative regulatory feedback loop when overexpressed in cells. Another explanation for this result is that PPP1R2 may regulate other proteins besides PP1 that oppose the effect of PP1 on centrosome number.

PP1 inhibits AURKA by dephosphorylating threonine 288 in the activation loop of AURKA [21]. As expected, co-overexpression of PP1 with AURKA reversed the accumulation of centrosomes seen when AURKA or PP1 were overexpressed individually (Fig. 1j). Our results are consistent with a previous report that AURKA and PP1 activities are regulated by a negative feedback loop. These results provide critical support for previous findings of Katayama [21], by demonstrating a biological consequence of this feedback loop.

The ability of PPP1R2 to inhibit PP1 phosphatase activity is dependent on the phosphorylation state of PPP1R2 [30, 38]. Glycogen synthase kinase-3β phosphorylates and inactivates PPP1R2, thereby activating PP1 [29, 36, 37]. Overexpression of the phosphomimetic mutant PPP1R2 R2E, but not the phosphonull mutant, caused supernumerary centrosomes to the same extent as wild-type PPP1R2 (Fig. 2g) and approaching the increase seen with PP1 alone (Fig. 1j). This is consistent with the inactivated phosphorylated form of PPP1R2 increasing activity of PP1 to increase centrosome number.

Increased expression of AURKA causes tetraploidization due to failed cytokinesis [6]. Likewise, increased expression of PPP1R2 caused increased cellular DNA content. One possibility for this result is that PPP1R2 participates in cytokinesis. PPP1R2’s role in cytokinesis is supported by the finding that PPP1R2 is found at the midbody (Fig. 4a, c) and that overexpression of its phosphomimetic as well as truncation mutants lengthened the central spindle and disrupted its structure (Figs. 6 and 7). Given that PPP1R2 interacts with both PP1 and AURKA, and has been reported to balance Aurora B and PP1 activity during cytokinesis, we propose that PPP1R2 coordinates the activity of kinases and PP1 to maintain central spindle architecture [23, 24, 39].

We demonstrate that both PPP1R2 and PP1 localized to the midbody and that PP1’s localization there is dependent on both N- and C-terminal domains of PPP1R2 (Fig. 4c). Notably, full length PPP1R2 enhanced PP1 localization at the midbody. The phosphomimetic mutant also increased PP1 localization supporting a role for PPP1R2 phosphorylation in targeting PP1 at cytokinesis. These data suggest that PPP1R2 targets PP1 to the midbody and this action relies on PPP1R2 phosphorylation. We propose that overexpression of PPP1R2 leads to an increase in phosphatase activity at the midbody resulting in less protein phosphorylation at the midbody with serious consequences to its function, as we observe here. During preparation of this manuscript, a study was published reporting the midbody interactome [33]. Results reported here expands the PP1 interactome to include PPP1R2. Further studies will be necessary to uncover the molecular mechanisms of PPP1R2’s effect on midbody structure through changes in activity of PP1 and AURKA at the centrosome.

PP1 regulates both phosphorylation of central spindle complexes [33] and cytokinesis by establishing proper recruitment of Cep55 and related abscission machinery to the midbody ring [12]. Aurora B Kinase (AURKB) has been shown to regulate midbody architecture and PPP1R2 has been shown to regulate AURKB at the midbody [24, 39]. Our data further demonstrates misshaped and elongated midbody structures during overexpression of both PPP1R2 phosphomimetic and truncation mutants (Figs. 6 and 7). Based on our data and previous reports regarding Aurora B kinase midbody regulation [24, 39], we propose PPP1R2 has a role in regulating kinase and phosphatase activity to control central spindle architecture and therefore cytokinesis. The mechanism whereby PPP1R2’s recruitment of PP1 to the midbody is involved in maintaining midbody architecture will be investigated in future studies.

PPP1R2 overexpression reduced phosphorylation of both AURKA and PP1 at the centrosome (Fig. 8c-d) independent of a change in global phosphorylation of either enzyme (Fig. 8b). These changes correlated with an increase in PP1 activity and a decrease in AURKA activity (Fig. 8a). Inhibition of AURKA by the C-terminus of PPP1R2 caused a stimulation of PP1 activity and a subsequent loss of AURKA and PP1 phosphorylation level at the centrosome (Fig. 8a). This is consistent with our proposal that inactivation of AURKA by PPP1R2 results in activation of PP1 at the centrosome. Changes in phosphorylation at the centrosome could have multiple effects on centrosome function including impaired protein recruitment and maintenance of the pericentriolar matrix [14, 20, 40]. Further studies will be necessary to investigate PPP1R2’s role in regulating the structure of the pericentriolar matrix and protein recruitment.

## Conclusions

Our data support the conclusion that PPP1R2 represents a new regulator of cytokinesis. This is consistent with both PPP1R2’s localization at the midbody and the increase in nuclear content when PPP1R2 is overexpressed. PPP1R2 overexpression increased PP1 localization at the midbody and this recruitment required both terminal domains on PPP1R2 necessary for binding to AURKA and PP1. Furthermore, both phosphorylation site and truncation mutants of PPP1R2 caused disruption in central spindle structure, both in length and integrity. We conclude that the increase in centrosome numbers we see after PPP1R2 overexpression is the result of incomplete cytokinesis caused by increased phosphatase activity at the midbody. In addition, we show that PPP1R2 interacts with AURKA and PP1 to regulate phosphorylation and activity of both AURKA and PP1 at the centrosome. Paradoxically, we find that overexpression of PPP1R2 increased PP1 activity rather than its well documented role as an inhibitor. Our study demonstrates that PPP1R2 activation of PP1 was dependent on PPP1R2’s C-terminus; the binding site for AURKA. Therefore, we propose that the increase in PP1 activity we observe is the indirect result of decreased activity of AURKA, an inhibitor of PP1 in PPP1R2 overexpressing cells. Increased phosphatase activity at the midbody would alter the mechanics of midbody assembly and abscission at cytokinesis.

## Methods

### Cell culture and DNA transfection

Human pigmented retinal epithelial cells (ARPE-19; American Type Tissue Collection) were grown in DMEM-F12 media supplemented with 10% fetal bovine serum and 1% penicillin-streptomycin. The PPP1R2 overexpressing plasmid was constructed by inserting the *Ppp1R2* coding sequence (a gift of Dr. Srinivasan Vijayaraghavan, Kent State University) in frame with the FLAG tag of the mammalian expression vector CMVFLAG 3X-14 (Sigma Aldrich). The PP1 plasmid was a gift of Dr. James McDonald, Western University, Cancer Research Center. The AURKA plasmid was obtained from Dr. Eric Nigg, University of Basil. PPP1R2 phospho-mutants were generated using the QuikChange® site-directed mutagenesis kit (Stratagene) and the PPP1R2 deletion mutants were obtained from Dr. David Brautigan, University of Virginia. ARPE-19 cells plated on glass coverslips were grown to approximately 70% confluence then grown for 24 h prior to transfection. Cells were transfected using Lipofectamine-2000 (Invitrogen) according to manufacturer’s recommendations.

### Immunofluorescence and measurement of protein localization

Transfected cells were fixed and permeabilized with methanol, then nonspecific binding was blocked by incubation in 3% BSA in TBST (20 mM Tris, pH 7.5, 150 mM NaCl, 2 mM EGTA, 0.1% Triton X-100) for 30 min. The cells were incubated overnight at 4 °C with primary antibody, then with secondary antibodies conjugated to Alexa Fluor-488 and 594 (1:200, Life Technologies). α-tubulin was detected with a goat polyclonal antibody (1:200, 74,010 clone TUBA4A, Life Sciences), PPP1R2 with a rabbit polyclonal antibody (1:100, 851,753, MyBioSource), pPPP1R2 with a rabbit polyclonal antibody (1:100, 44-1160G, Thermo Fisher Scientific), AURKA with a rabbit polyclonal antibody (1:100, PA5–34700,Thermo Fisher Scientific), pAURKA rabbit polyclonal antibody (1:100, 44-1210G, Thermo Fisher Scientific), PP1 with a rabbit polyclonal antibody (1:100, L5–454752, Lifespan Biosciences), pPP1 rabbit polyclonal antibody (1:100, PA5–17819, Thermo Fisher Scientific), FLAG with a rabbit polyclonal antibody (1:500, PA1984B; Thermo Fisher Scientific), c-myc with a rabbit polyclonal antibody (1:500, NB600–335, Novus Biologicals), and γ-tubulin with a mouse monoclonal antibody (1:50, PA5–34815, Thermo Fisher Scientific). DNA was labeled with Vectashield mounting media containing 4′, 6-diamidino-2-phenylindole (DAPI) dye (Vector Laboratories).

The intracellular localization of proteins was visualized using a Nikon E600 fluorescence microscope, Pan Fluor 100X objective (N.A. 0.5–1.3) or Pan Fluor 40X objective (N.A. 0.75), fit with appropriate filters. Images were captured with an Orca II CCD camera (Hamamatsu) and Metamorph image analysis and acquisition software (Universal Imaging Corporation). Images were exported to ImageJ (NIH) and only linear adjustments to brightness and/or contrast were performed.

Midbody protein localization was quantified using Metamorph software. Parameters were set by using 10 × 10-pixel sized squares with each having an area of 0.87 μm^2^. Ten squares were aligned across the midbody at intervals of 1 μm starting from the midbody as a central orienting landmark.

### DNA content measurement

Cells were transfected with FLAG, PPP1R2, or AURKA plasmids, fixed with 70% ethanol, and stained with propidium iodide. DNA content was assessed using a cell flow cytometer (Becton Dickinson FACScan Cytometer) and CellQuest (BD biosciences) software. Cell counts were capped at 3000 for each run and parameters set to exclude all doublet cells and cells with expected DNA content. Remaining polyploid cells were counted and divided by total cell count to calculate percentage of cells with abnormal ploidy levels.

### Cell lysate preparation and ELISA

Transfected ARPE cells were collected using a cell lifter and solubilized in cell lysis buffer (50 mM Tris, 1 mM EGTA, 1% NP40). Cell lysate was treated with 1:100 Halt phosphatase inhibitor cocktail (Sigma-Aldrich), 1:10 protease inhibitor cocktail (Calbiochem) and lysed using a freeze-thaw method using liquid nitrogen. Following freeze-thaw, benzonase nuclease (Novagen) digested nuclear material in the lysate. Protein concentration of the lysate was measured using a Pierce BCA protein assay kit (Thermofisher).

96-well plates (Falcon) were coated with cell lysate at a protein concentration of 20μg/ml; 1 μg of protein per well. Plates were blocked with 3% BSA solution diluted in phosphate buffer buffered saline (PBS, 13.7 mM NaCl, 2.7 mM KCl, 1.4 mM KH_2_PO_4_, 4.3 mM Na_2_HPO_4_). Plates were then incubated at 4^ο^C overnight and then washed with 1x PBS. Cells were then incubated with primary antibodies targeted to FLAG (1:500, F1804, Sigma Aldrich), AURKA (1:500, IHC-00062, Thermofisher), PP1 (1:500, A-300-904A-M Thermofisher), pPP1 (1:500, 25,815, Cell Signaling) at 4^ο^C overnight. Cells were washed and incubated with donkey anti-rat (1:2000, Jackson Immuno Research Lab) and donkey anti-mouse (1:2000, Jackson Immuno Research Lab) HRP conjugated secondary antibodies for 1 hour at room temperature. After a final wash, bound antibody was detected with o-phenylenediamine dihydrochloride (OPD) 1 g/L in buffer (0.05 M citric acid, 0.05 M sodium phosphate, 1% hydrogen peroxide, pH 5) for 30 minutes at 37^ο^C. Fluorescence was then measured using a plate reader fitted with a 450 nm filter.

### AURKA immunoprecipitation and activity assay, phosphatase activity assay

Protein complexes were collected by immunoprecipitation using Sepharose bead-antibody capture. Briefly, affinity purified antibody to AURKA (1:500, IHC-00062, Thermofisher) was incubated with precleared cell lysate (> 1 mg protein) followed by anti-rabbit IgG beads. Immunoprecipated proteins were detected by ELISA with anti-AURKA antibody (1:500, IHC-00062, Thermofisher), and anti-HRP (1:2000, 131,366 Abcam). Negative control for coimmunoprecipitation was a sample without antibody.

AURKA activity was detected using the Universal Kinase Assay Kit on samples obtained from AURKA immunoprecipitation (abcam, ab138879). Samples were prepped and processed through the kinase assay kit as per manufacturer’s instructions. Samples were measured by plate reader at 540/590 nm excitation/emission.

PP1 activity was detected using a RediPlate™ 96 EnzChek® Serine/Threonine Phosphatase Assay Kit as per manufacturer’s instruction. Cell lysate was prepared following transfection and phosphatase activity in equal amounts of protein measured by plate reader at 355/460 nm excitation/emission.

### Statistical analyses

The data for centrosome quantitation was expressed as mean ± SEM. The differences between groups were analyzed using a One-way ANOVA and unpaired Student’s t test with JMP Version 13.1. Differences at *p* ≤ 0.05 were considered statistically significant. We used the software Q*Power to perform calculate sample sizes appropriate for 80% power and an alpha value below 0.2.

## Supplementary Information

**Additional file 1.** Expression of ectopically expressed tagged proteins. Fusion proteins tagged with either FLAG (black) or myc (blue) were detected in cells by ELISA 24 h after transfection.

## Data Availability

The datasets used and/or analyzed during the current study are available from the corresponding author on reasonable request.

## Abbreviations

AURKA: Aurora kinase A
PP1: Protein phosphatase 1
PPP1R2: Protein phosphatase 1 regulatory subunit 2
NEK2: Never in mitosis kinase 2
AURKB: Aurora kinase B
DMEM: Dulbecco’s modified eagle media
CMV-FLAG: Cytomegalovirus FLAG
TBST: Tris buffered saline with tween
CCD: Charge coupled devise
FACS: Fluorescence activated cell sorting
PBS: Phosphate buffered saline
HRP: Horse radish peroxidase
ELISA: Enzyme-linked immunosorbent assay
ANOVA: Analysis of variance

## References

[1] Fujita H, Yoshino Y, Chiba N (2016). Regulation of the centrosome cycle. Mol Cell Oncol.

[2] Khodjakov A, Rieder CL (2001). Centrosomes enhance the fidelity of cytokinesis in vertebrates and are required for cell cycle progression. J Cell Biol.

[3] Eggert US, Mitchison TJ, Field CM (2006). Animal cytokinesis: from parts list to mechanisms. Annu Rev Biochem.

[4] Ganem NJ, Storchova Z, Pellman D (2007). Tetraploidy, aneuploidy and cancer. Curr Opin Genet Dev.

[5] Anand S, Penrhyn-Lowe S, Venkitaraman AR (2003). AURORA-A amplification overrides the mitotic spindle assembly checkpoint, inducing resistance to Taxol. Cancer Cell.

[6] Meraldi P, Honda R, Nigg EA (2002). Aurora-a overexpression reveals tetraploidization as a major route to centrosome amplification in p53−/− cells. EMBO J.

[7] Levine MS, Bakker B, Boeckx B, Moyett J, Lu J, Vitre B, Spierings DC, Lansdorp PM, Cleveland DW, Lambrechts D (2017). Centrosome amplification is sufficient to promote spontaneoustumorigenesis in mammals. Dev Cell.

[8] Rivera-Rivera Y, Saavedra HI (2016). Centrosome - a promising anti-cancer target. Biologics.

[9] Moura M, Conde C. Phosphatases in Mitosis: Roles and Regulation. Biomolecules. 2019;9(2):55. 10.3390/biom9020055 PMC6406801 30736436

[10] Bhowmick R, Thakur RS, Venegas AB, Liu Y, Nilsson J, Barisic M, Hickson ID. The RIF1-PP1 axis controls abscission timing in human cells. Curr Biol. 2019;29(7):1232–42.e5. 10.1016/j.cub.2019.02.037 30905608

[11] Ceulemans H, Bollen M (2004). Functional diversity of protein phosphatase-1, a cellular economizer and reset button. Physiol Rev.

[12] Gao K, Zhang Y, Shi Q, Zhang J, Zhang L, Sun H, Jiao D, Zhao X, Tao H, Wei Y (2018). iASPP-PP1 complex is required for cytokinetic abscission by controlling CEP55 dephosphorylation. Cell Death Dis.

[13] Bollen M, Peti W, Ragusa MJ, Beullens M (2010). The extended PP1 toolkit: designed to create specificity. Trends Biochem Sci.

[14] Nasa I, Trinkle-Mulcahy L, Douglas P, Chaudhuri S, Lees-Miller SP, Lee KS, Moorhead GB (2017). Recruitment of PP1 to the centrosomal scaffold protein CEP192. Biochem Biophys Res Commun.

[15] Carmena M, Earnshaw WC (2003). The cellular geography of aurora kinases. Nat Rev Mol Cell Biol.

[16] Glover DM, Leibowitz MH, McLean DA, Parry H (1995). Mutations in aurora prevent centrosome separation leading to the formation of monopolar spindles. Cell.

[17] Giet R, McLean D, Descamps S, Lee MJ, Raff JW, Prigent C, Glover DM (2002). Drosophila Aurora a kinase is required to localize D-TACC to centrosomes and to regulate astral microtubules. J Cell Biol.

[18] Karthigeyan D, Prasad SB, Shandilya J, Agrawal S, Kundu TK (2011). Biology of Aurora a kinase: implications in cancer manifestation and therapy. Med Res Rev.

[19] Nikonova AS, Astsaturov I, Serebriiskii IG, Dunbrack RL, Golemis EA (2013). Aurora a kinase (AURKA) in normal and pathological cell division. Cell Mol Life Sci.

[20] Zhou H, Kuang J, Zhong L, Kuo WL, Gray JW, Sahin A, Brinkley BR, Sen S (1998). Tumour amplified kinase STK15/BTAK induces centrosome amplification, aneuploidy and transformation. Nat Genet.

[21] Katayama H, Zhou H, Li Q, Tatsuka M, Sen S (2001). Interaction and feedback regulation between STK15/BTAK/Aurora-a kinase and protein phosphatase 1 through mitotic cell division cycle. J Biol Chem.

[22] Eto M, Elliott E, Prickett TD, Brautigan DL (2002). Inhibitor-2 regulates protein phosphatase-1 complexed with NimA-related kinase to induce centrosome separation. J Biol Chem.

[23] Satinover DL, Leach CA, Stukenberg PT, Brautigan DL (2004). Activation of Aurora-a kinase by protein phosphatase inhibitor-2, a bifunctional signaling protein. Proc Natl Acad Sci U S A.

[24] Wang W, Stukenberg PT, Brautigan DL (2008). Phosphatase inhibitor-2 balances protein phosphatase 1 and aurora B kinase for chromosome segregation and cytokinesis in human retinal epithelial cells. Mol Biol Cell.

[25] Lioutas A, Vernos I (2013). Aurora a kinase and its substrate TACC3 are required for central spindle assembly. EMBO Rep.

[26] Meraldi P, Nigg EA (2001). Centrosome cohesion is regulated by a balance of kinase and phosphatase activities. J Cell Sci.

[27] Helps NR, Luo X, Barker HM, Cohen PT (2000). NIMA-related kinase 2 (Nek2), a cell-cycle-regulated protein kinase localized to centrosomes, is complexed to protein phosphatase 1. Biochem J.

[28] Connor JH, Frederick D, Huang H, Yang J, Helps NR, Cohen PT, Nairn AC, DePaoli-Roach A, Tatchell K, Shenolikar S (2000). Cellular mechanisms regulating protein phosphatase-1. A key functional interaction between inhibitor-2 and the type 1 protein phosphatase catalytic subunit. J Biol Chem.

[29] Holmes CF, Campbell DG, Caudwell FB, Aitken A, Cohen P (1986). The protein phosphatases involved in cellular regulation. Primary structure of inhibitor-2 from rabbit skeletal muscle. Eur J Biochem.

[30] Satinover DL, Brautigan DL, Stukenberg PT (2006). Aurora-a kinase and inhibitor-2 regulate the cyclin threshold for mitotic entry in Xenopus early embryonic cell cycles. Cell Cycle.

[31] Brautigan DL, Sunwoo J, Labbe JC, Fernandez A, Lamb NJ (1990). Cell cycle oscillation of phosphatase inhibitor-2 in rat fibroblasts coincident with p34cdc2 restriction. Nature.

[32] Mi J, Guo C, Brautigan DL, Larner JM (2007). Protein phosphatase-1alpha regulates centrosome splitting through Nek2. Cancer Res.

[33] Capalbo L, Bassi ZI, Geymonat M, Todesca S, Copoiu L, Enright AJ, Callaini G, Riparbelli MG, Yu L, Choudhary JS (2019). The midbody interactome reveals unexpected roles for PP1 phosphatases in cytokinesis. Nat Commun.

[34] Marumoto T, Zhang D, Saya H (2005). Aurora-a - a guardian of poles. Nat Rev Cancer.

[35] Mangal S, Sacher J, Kim T, Osorio DS, Motegi F, Carvalho AX, Oegema K, Zanin E (2018). TPXL-1 activates Aurora a to clear contractile ring components from the polar cortex during cytokinesis. J Cell Biol.

[36] Cohen P (1989). The structure and regulation of protein phosphatases. Annu Rev Biochem.

[37] Sakashita G, Shima H, Komatsu M, Urano T, Kikuchi A, Kikuchi K (2003). Regulation of type 1 protein phosphatase/inhibitor-2 complex by glycogen synthase kinase-3beta in intact cells. J Biochem (Tokyo).

[38] Puntoni F, Villa-Moruzzi E (1995). Phosphorylation of the inhibitor-2 of protein phosphatase-1 by cdc2-cyclin B and GSK3. Biochem Biophys Res Commun.

[39] McKenzie C, Bassi ZI, Debski J, Gottardo M, Callaini G, Dadlez M, D'Avino PP. Cross-regulation between Aurora B and Citron kinase controls midbody architecture in cytokinesis. Open Biol. 2016;6(3). 10.1098/rsob.160019 PMC4821246 27009191

[40] Meng L, Park JE, Kim TS, Lee EH, Park SY, Zhou M, Bang JK, Lee KS (2015). Bimodal interaction of mammalian polo-like kinase 1 and a Centrosomal scaffold, Cep192, in the regulation of bipolar spindle formation. Mol Cell Biol.

