# Supplementary material for: The PP1 regulator PPP1R2 coordinately regulates AURKA and PP1 to control centrosome phosphorylation and maintain central spindle architecture
Source: BMC Mol Cell Biol. 2020 Nov 25;21:84. doi: 10.1186/s12860-020-00327-5 (PMC7687763; doi:10.1186/s12860-020-00327-5)
Supplement: Supplementary file 1 — Additional file 1. Expression of ectopically expressed tagged proteins. Fusion proteins tagged with either FLAG (black) or myc (blue) were detected in cells by ELISA 24 h after transfection. [file 12860_2020_327_MOESM1_ESM.pptx]

## Slide 1
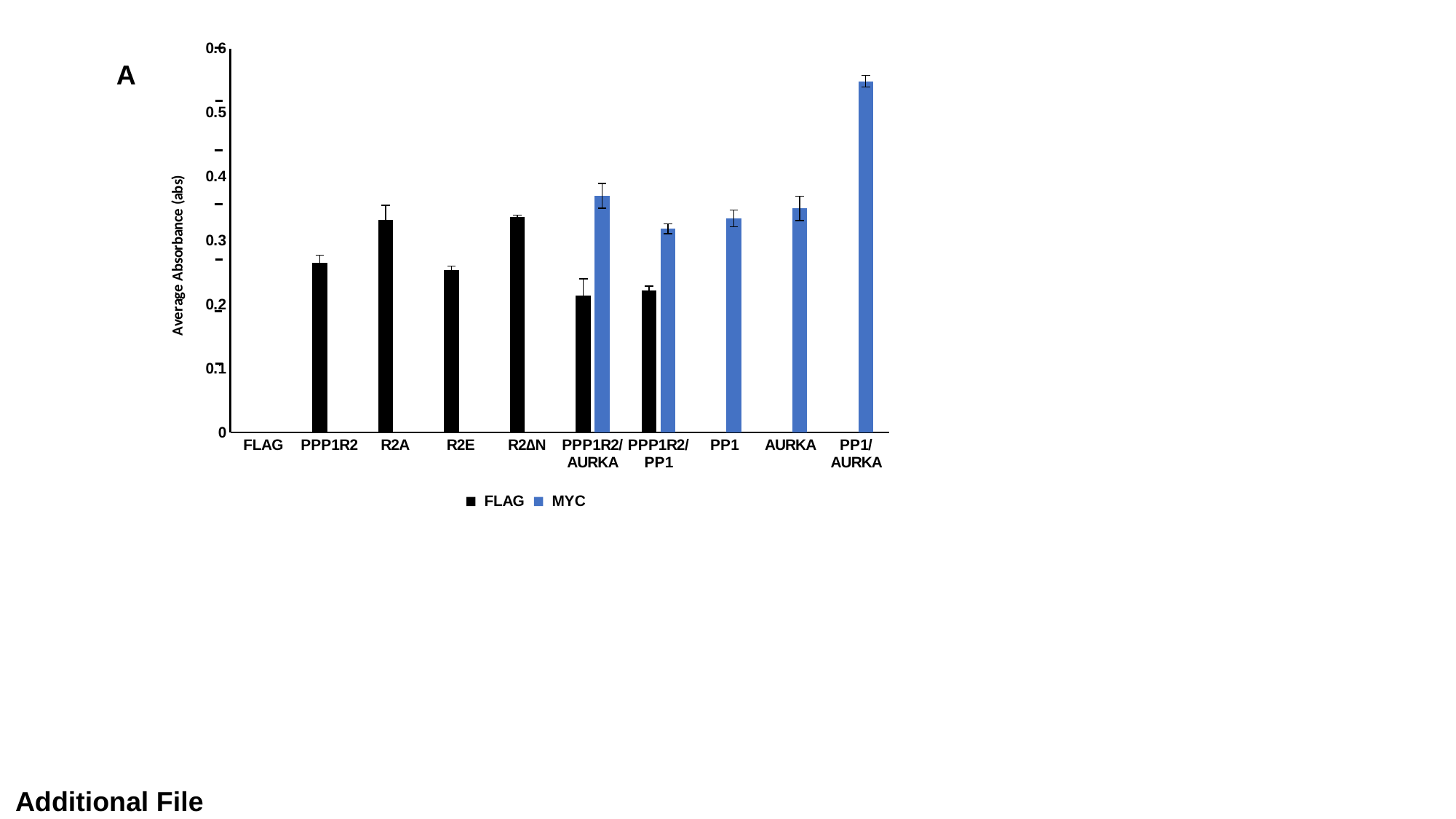

### Chart
| Category | FLAG | MYC |
|---|---|---|
| FLAG | 0.0 | 0.0 |
| PPP1R2 | 0.265 | 0.0 |
| R2A | 0.33199999999999996 | 0.0 |
| R2E | 0.25366666666666665 | 0.0 |
| R2∆N | 0.337 | 0.0 |
| PPP1R2/AURKA | 0.21366666666666664 | 0.3696666666666667 |
| PPP1R2/PP1 | 0.222 | 0.3186666666666667 |
| PP1 | 0.0 | 0.3346666666666667 |
| AURKA | 0.0 | 0.35033333333333333 |
| PP1/AURKA | 0.0 | 0.5489999999999999 |A
Additional File
